# Curdepsidone A Induces Intrinsic Apoptosis and Inhibits Protective Autophagy via the ROS/PI3K/AKT Signaling Pathway in HeLa Cells

**DOI:** 10.3390/md22050227

**Published:** 2024-05-17

**Authors:** Sunjie Xu, Zhimin Li, Xiujuan Xin, Faliang An

**Affiliations:** 1 State Key Laboratory of Bioreactor Engineering, East China University of Science and Technology, 130 Mei Long Road, Shanghai 200237, China; y20180125@mail.ecust.edu.cn (S.X.); lizm@ecust.edu.cn (Z.L.); xinxj@ecust.edu.cn (X.X.); 2 Marine Biomedical Science and Technology Innovation Platform of Lin-Gang Special Area, No. 4, Lane 218, Haiji Sixth Road, Shanghai 201306, China

**Keywords:** curdepsidone A, HeLa cells, anti-tumor activity, ROS, PI3K/AKT signaling pathway

## Abstract

Among female oncology patients, cervical cancer stands as the fourth most prevalent malignancy, exerting significant impacts on their health. Over 600,000 women received the diagnosis of cervical cancer in 2020, and the illness claimed over 300,000 lives globally. Curdepsidone A, a derivative of depsidone, was isolated from the secondary metabolites of *Curvularia* sp. IFB-Z10. In this study, we revised the molecular structure of curdepsidone A and investigated the fundamental mechanism of the anti-tumor activity of curdepsidone A in HeLa cells for the first time. The results demonstrated that curdepsidone A caused G0/G1 phase arrest, triggered apoptosis via a mitochondrial apoptotic pathway, blocked the autophagic flux, suppressed the PI3K/AKT pathway, and increased the accumulation of reactive oxygen species (ROS) in HeLa cells. Furthermore, the PI3K inhibitor (LY294002) promoted apoptosis induced by curdepsidone A, while the PI3K agonist (IGF-1) eliminated such an effect. ROS scavenger (NAC) reduced curdepsidone A-induced cell apoptosis and the suppression of autophagy and the PI3K/AKT pathway. In conclusion, our results revealed that curdepsidone A hindered cell growth by causing cell cycle arrest, and promoted cell apoptosis by inhibiting autophagy and the ROS-mediated PI3K/AKT pathway. This study provides a molecular basis for the development of curdepsidone A as a new chemotherapy drug for cervical cancer.

## 1. Introduction

Despite being highly preventable, cervical cancer continues to afflict over 600,000 women worldwide, with more than 300,000 succumbing to the disease. Especially in less-developed regions, cervical cancer remains a primary contributor to cancer-related mortality [1]. Although the current treatment of cervical cancer via a combination of chemotherapy, surgery, and radiotherapy shows promising effects, the severe side effects of such treatment may lead to therapeutic failure [2,3,4]. Therefore, research into novel compounds that are less likely to cause side effects and help to treat cervical cancer is imperative [3], and marine-derived drugs may be an effective way to address this medical challenge.

Many marine natural products, including alkaloids, polyketides, peptides, and terpenes, tend to have significant biological activities [5,6,7]. As of 2022, twenty drugs derived from the ocean have been clinically used, with the majority employed in cancer therapy [8]. The One Strain, Many Compounds (OSMAC) strategy induces the production of different secondary metabolites by modulating the culture conditions of microorganisms, which helps to isolate and obtain novel compounds with good biological activities [9,10]. Depsidones are a class of polyphenolic polyketides formed via the oxidative coupling of esters of two polyketide benzoic acid derivatives [11]. They exhibit a variety of bioactivities such as cytotoxic [12,13], anti-inflammatory [14,15], antimicrobial [13,16,17], antimalarial [18], antioxidant [19], and antidiabetic [20] activities. Curdepsidones A-G, derivatives of depsidone, were first isolated from the secondary metabolites of a marine white croaker-associated fungus *Curvularia* sp. IFB-Z10 in our group [21,22]. Moreover, curdepsidone A was identified to have significant cytotoxicity against human hepatoma cells in vitro [21]. However, whether the anti-tumor activity of curdepsidone A is broad-spectrum and its anti-tumor mechanism remain unclear.

Apoptosis and cell cycle arrest are both essential mechanisms underlying the anticancer effects of drugs. The main reason for the uncontrolled proliferation of tumor cells is the disruption of the cell cycle. Therefore, the regulation of the cell cycle stands as a pivotal strategy for restraining tumor cell growth [23]. The primary mechanism of tumor cell death is apoptosis, a type of spontaneous cell death promoted by caspase-related proteins and controlled by multiple signaling pathways [24]. Besides apoptosis, natural products have the potential to preferentially affect cancer cells by targeting cellular autophagy, which is one of the current ideas in anticancer drug development [25]. Autophagy, a cellular self-digestion process, is used to get rid of misfolded proteins and aging, damaged organelles [26]. Autophagy is widely regarded as possessing a dual role in cancer, acting as both a tumor suppressor and a facilitator of tumor growth [27]. The PI3K/AKT pathway, which is anomalously activated in human tumor cells, exerts a pivotal role in autophagy regulation [28] and is closely associated with cell growth, survival, and metabolism [29,30]. Moreover, the PI3K/AKT pathway and autophagy are strongly associated with elevated reactive oxygen species (ROS) levels [27,28]. ROS often causes irreversible damage to mitochondrial function and DNA, suppresses the growth of tumors, and triggers the apoptosis of tumor cells [31,32]. Therefore, both ROS and the PI3K/AKT pathway are important targets in cancer therapy.

In this study, we first revised the structure of curdepsidone A through skeleton carbon calculation with DP4 probability. Further studies considered that curdepsidone A can also suppress the growth of other tumor cells. The objective of this study was to assess the anticancer properties of curdepsidone A in human cervical cancer HeLa cells. We investigated the role of cell cycle, apoptosis, autophagy, ROS generation, and the PI3K/AKT pathway in the growth inhibition of HeLa cells produced by curdepsidone A. We found that curdepsidone A could inhibit HeLa cell viability by causing cell cycle arrest, inducing apoptosis, and suppressing protective autophagy and the ROS-mediated PI3K/AKT pathway. These results indicated that curdepsidone A may be a potential marine anti-tumor drug candidate.

## 2. Results

### 2.1. Revision of the Molecular Structure of Curdepsidone A

**Figure 1:**
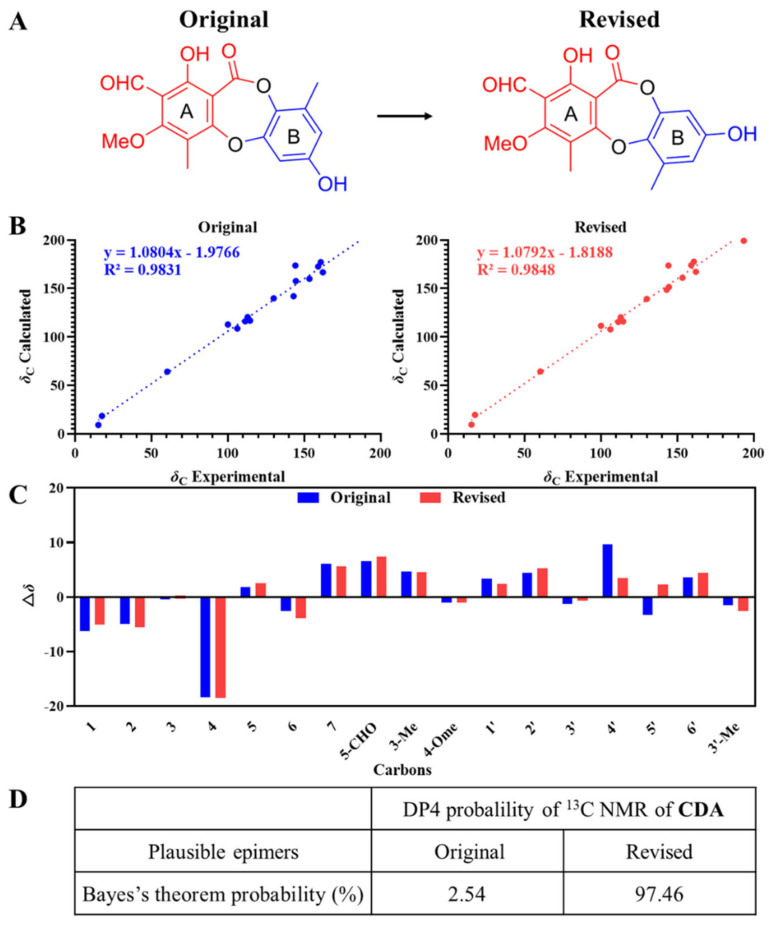
Revision of the molecular structure of curdepsidone A (CDA). (**A**) The molecular structure of curdepsidone A. (**B**) The plot of linear correlation between the calculated and actual values for the ^13^C of curdepsidone A. (**C**) Relative error analysis performed to assess the deviation between the calculated and actual ^13^C values. (**D**) DP4 probability analysis was employed to evaluate the confidence level associated with the assignment of ^13^C values for curdepsidone A.

Curdepsidone A, a white powder with the molecular formula C_17_H_14_O_7_, was obtained from the crude culture extracts of *Curvularia* sp. IFB-Z10 in our group in 2018 with the OSMAC strategy [21]. Its structure was determined only using HRESIMS and NMR data. However, there was still an overlooked point of structure determination, which was also proved and revised in the project of separating and purifying curdepsidone analogues by our group [22]. The qccNMR technique was employed to determine the connection between the structural units A and B within these compounds, revealing the presence of an ester linkage positioned between C-2 and C-1′. Subsequently, to validate the planar configuration of curdepsidone A, a DP4 carbon calculation was conducted (Figure 1A,B), and the position of substituents on the B unit within curdepsidone A was revised according to the Bayes theorem probability value of 97.46%, executing a 180° rotation of the B unit in the vertical direction (Figure 1C).

### 2.2. Curdepsidone A Inhibited HeLa Cell Viability and Proliferation

**Figure 2:**
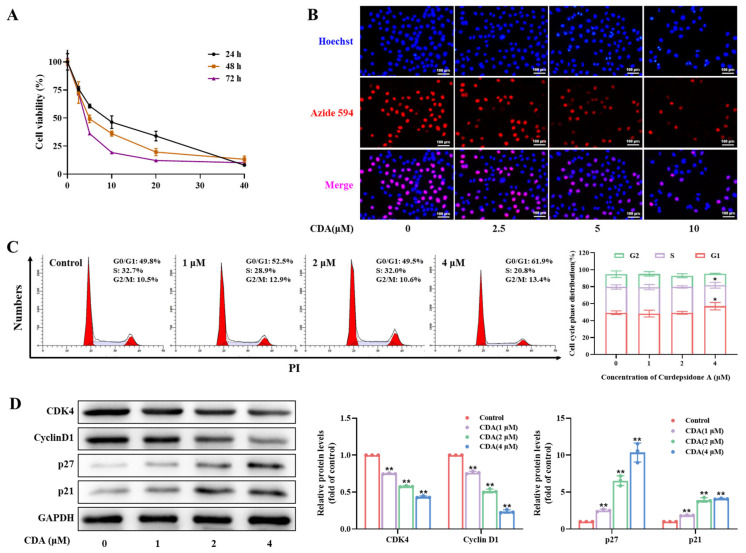
Curdepsidone A inhibited cell viability and induced G0/G1 phase arrest in HeLa cells. (**A**) HeLa cells were exposed to curdepsidone A at various concentrations for 24, 48, or 72 h. The cytotoxic activity of curdepsidone A in HeLa cell lines was measured. (**B**) HeLa cells were exposed to curdepsidone A for 24 h and stained with Hoechst 33342. DNA synthesis in HeLa cells was detected using an EdU assay. (**C**) HeLa cells were exposed to curdepsidone A for 24 h. The cell cycle in HeLa cells was analyzed. (**D**) The effects of curdepsidone A on the expression of the cell-cycle-related proteins CDK4, Cyclin D1, p27, and p21 at concentrations of 0, 1, 2, and 4 μM were examined. Values are mean ± SD, *n* = 3, * *p* < 0.05. ** *p* < 0.01.

The effects of curdepsidone A on the viability of seven types of tumor cell lines (Hela, Bel-7402, HepG2, K562, SW1116, MCF-7, and MGC803) were investigated in vitro using the MTT assay. Curdepsidone A inhibited the activity of different tumor cells, and human cervical cancer HeLa cells were the most sensitive to curdepsidone A among all the tested cells (Table 1, Figure S2). A variety of doses of curdepsidone A were used to treat HeLa cells. The outcomes demonstrated that curdepsidone A decreased HeLa cell viability in a dose- and time-dependent manner (Figure 2A). The half inhibitory concentration (IC_50_) value of curdepsidone A in HeLa cells at 48 h was 6.28 ± 0.38 μM. The EdU assay was used to identify DNA synthesis in cells to assess the impact of curdepsidone A on the proliferation of HeLa cells. The red spots in Figure 2B considerably decreased as the concentration of curdepsidone A increased, indicating that curdepsidone A suppressed HeLa cell proliferation in a dose-dependent manner.

### 2.3. Curdepsidone A Induced G0/G1 Phase Arrest in HeLa Cells

We examined the effect of curdepsidone A on cell cycle progression to explore the mechanism underlying the inhibition of cell growth. HeLa cells were exposed to 0, 1, 2, and 4 μM of curdepsidone A for 24 h. Afterward, flow cytometry was used to identify the number of cells in the cell cycle. Curdepsidone A enhanced the proportion of cells from 49.8% to 61.9% in the G0/G1 phase at a concentration of 4 μM, as Figure 2C shows. Additionally, curdepsidone A downregulated the levels of CDK4 and cyclin D1 and upregulated the levels of p27 and p21 (Figure 2D). All these findings suggested that treatment with curdepsidone A could result in cell cycle arrest at the G0/G1 phase, which, in turn, prevented HeLa cells from proliferating.

### 2.4. Curdepsidone A Induced Apoptosis of HeLa Cells

**Figure 3:**
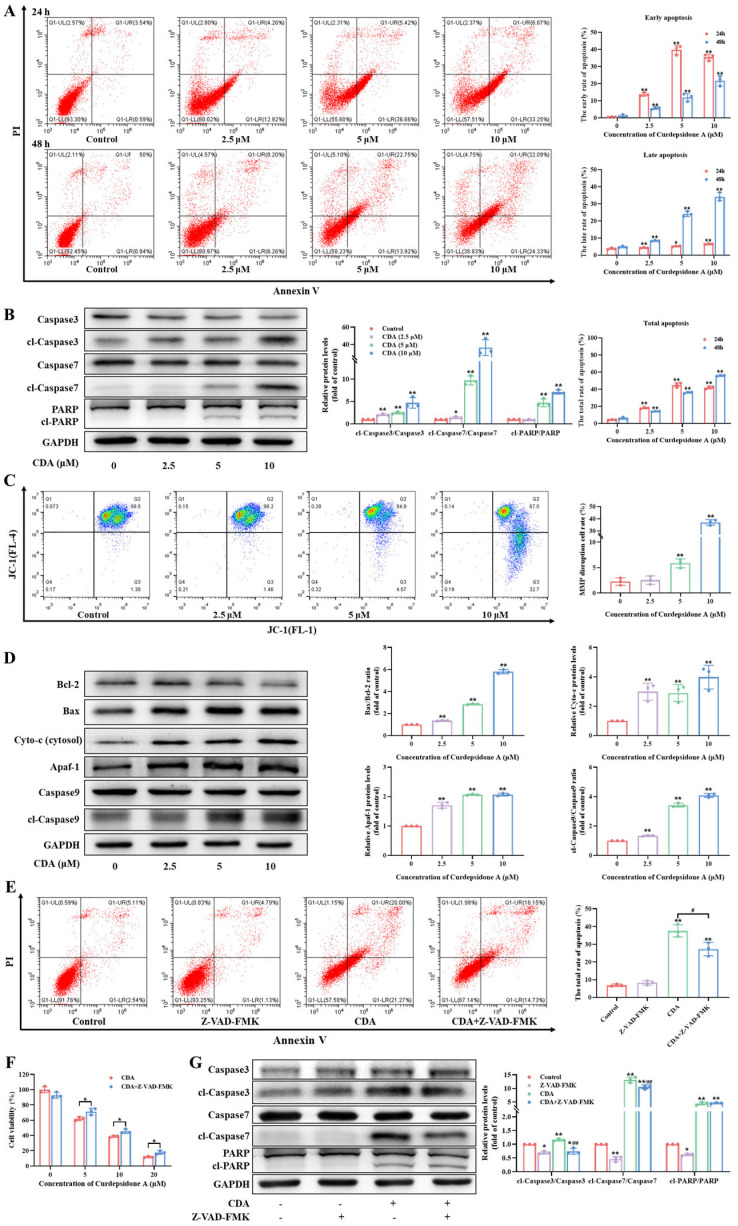
Curdepsidone A induced apoptosis of HeLa cells. (**A**) HeLa cells were exposed to curdepsidone A for 24 or 48 h. The effect of curdepsidone A on the apoptosis of HeLa cells was measured. (**B**) The effects of curdepsidone A on the expression of the apoptosis-related proteins caspase3, cl-caspase3, caspase7, cl-caspase7, PARP, and cl-PARP were examined. (**C**) Mitochondrial membrane potential (MMP) in HeLa cells treated with curdepsidone A for 48 h was analyzed. (**D**) The effects of curdepsidone A on the expression of the endogenous apoptosis-related proteins Bax, Bcl-2, Cyto-C (cytosol), Apaf-1, caspase9, and cl-caspase9 were examined. (**E**–**G**) HeLa cells were pretreated with Z-VAD-FMK (20 μM) for 2 h and then treated with curdepsidone A (10 μM) for 48 h. The apoptosis of HeLa cells (**E**), the cell viability (**F**), and the expression of caspase3, cl-caspase3, caspase7, cl-caspase7, PARP, and cl-PARP (**G**) were examined. Values are mean ± SD, *n* = 3, * *p* < 0.05, ** *p* < 0.01 versus the control group; ^#^ *p* < 0.05, ^##^ *p* < 0.01 versus the CDA group.

Both cell cycle arrest and apoptosis are key processes that inhibit cell viability. To explore the impact of curdepsidone A on HeLa cell apoptosis, we used flow cytometry to quantify the total rate of apoptosis in curdepsidone A-treated HeLa cells via double staining with Annexin V-FITC/PI. Compared with untreated cells, exposure to 10 μM of curdepsidone A for 24 h and 48 h increased the apoptosis cell percentage from 4.00% to 42.95% and from 5.04% to 51.04%, respectively, in a dose-dependent manner (Figure 3A). Moreover, after a 24-h treatment, HeLa cells were almost in the early apoptotic stage. Conversely, cells treated for 48 h were mostly in the late apoptotic stage. In addition, HeLa cells showed intense blue fluorescence when stained with Hoechst 33258, indicating nuclear condensation and DNA breakage (Figure 2B). Together, all these results showed that curdepsidone A caused HeLa cells to undergo apoptosis.

### 2.5. Curdepsidone A Induced Apoptosis via the Mitochondria Pathway

Mitochondrial membrane potential (MMP) will obviously decrease with the activation of the mitochondrial apoptosis pathway. JC-1 was used as an indicator to detect the changes in MMP. The findings showed that curdepsidone A caused a dose-dependent decrease in MMP (Figure 3C). In order to further validate apoptotic cell death, we assessed the apoptosis-related protein expressions of Bax; Bcl-2; cytochrome c (cyto-c); cl-caspase3, 7, and 9; and cl-PARP (Figure 3B,D). The expressions of cl-caspase3, 7, and 9; cl-PARP; Bax; and cytosolic cyto-c levels were significantly elevated via curdepsidone A treatment. Meanwhile, the expression level of the anti-apoptosis protein Bcl-2 declined. Furthermore, curdepsidone A-induced apoptotic cell death was significantly reduced by Z-VAD-FMK, a pan-caspase inhibitor (Figure 3E). Collectively, these findings indicated that curdepsidone A caused HeLa cells to undergo endogenous apoptosis through the mitochondrial pathway.

### 2.6. Curdepsidone A Inhibited Protective Autophagy in HeLa Cells

**Figure 4:**
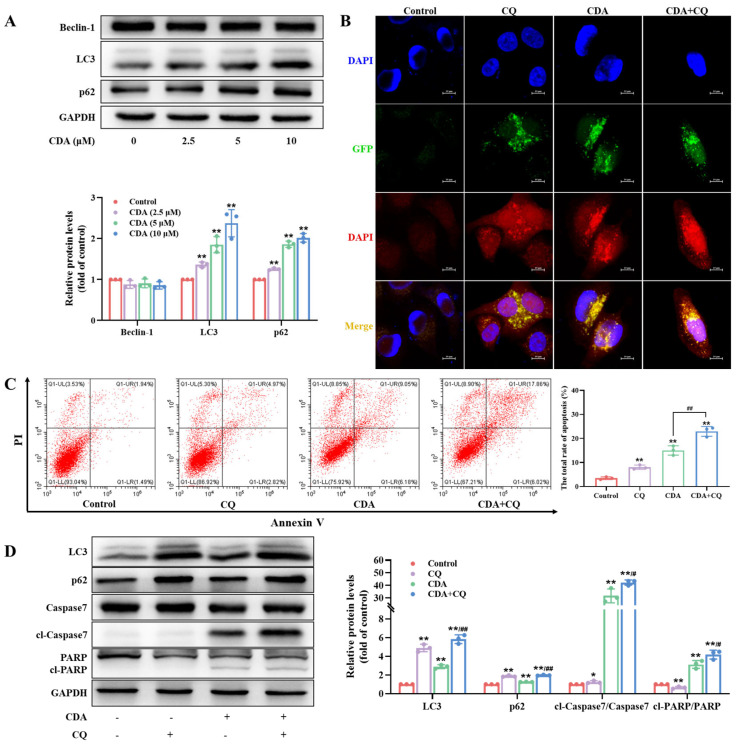
Curdepsidone A inhibited protective autophagy in HeLa cells. (**A**) HeLa cells were exposed to curdepsidone A for 48 h. The expression of autophagy-related proteins was examined. (**B**) The autophagy flux was detected using GFP-RFP-LC3B double-labeled adenovirus. (**C**,**D**) HeLa cells were treated with curdepsidone A (10 μM) alone or in combination with CQ (10 μM) for 48 h. The apoptosis of HeLa cells (**C**) and the expression of LC3, p62, caspase7, cl-caspase7, PARP, and cl-PARP (**D**) were examined. Values are mean ± standard deviation, *n* = 3, * *p* < 0.05, ** *p* < 0.01 versus the control group; *^#^ p* < 0.05, ^##^ *p* < 0.01 versus the CDA group.

In consideration of autophagy being an internal defense system that enables cells to withstand a variety of stresses, it is commonly recognized that autophagy plays a critical role in the advancement of cancer. To investigate whether curdepsidone A affected autophagy in HeLa cells, we explored the expression of autophagy-related proteins. The findings revealed that while curdepsidone A did not affect the expression of Beclin-1, it increased intracellular LC3 and p62 accumulation in HeLa cells (Figure 4A). An effective method to differentiate the balance between autophagosomes and autolysosomes throughout autophagic development is to count the amount of GFP-RFP-LC3B puncta [33]. When GFP fluorescent protein is in an acidic environment, its fluorescence signal tends to quench. Since lysosomes are acidic, autolysosomes appear as red spots. On the other hand, autophagosomes are in a neutral environment; hence, they display as yellow spots. Moreover, CQ can inhibit the formation of autolysosomes [34]. Therefore, GFP-RFP-LC3B puncta and CQ were adopted to investigate the effect of curdepsidone A on the process of autophagic flux. As shown in Figure 4B, similar to the effects of the CQ treatment, curdepsidone A treatment alone increased yellow spots in HeLa cells. When curdepsidone A was administered in combination with CQ (5 μM) to assess the effect of autophagy on apoptosis, the results demonstrated that the addition of CQ with curdepsidone A markedly raised the ratio of apoptotic cells (Figure 4C) and promoted the cleavage of caspase7 and PARP compared to groups with curdepsidone A alone on HeLa cells (Figure 4D). The combination of curdepsidone A and CQ might promote the apoptosis of HeLa cells by enhancing autophagy flux blocking. The results showed that curdepsidone A obstructed autophagy flux during the autolysosome formation stage. The accumulation of autophagosomes resulting from the blockage of autophagy flux was deleterious to cell growth and could induce apoptosis in HeLa cells. It could be deduced that curdepsidone A might inhibit protective autophagy in HeLa cells, which could promote the occurrence of apoptosis.

### 2.7. Curdepsidone A Suppressed the PI3K/AKT Pathway in HeLa Cells

**Figure 5:**
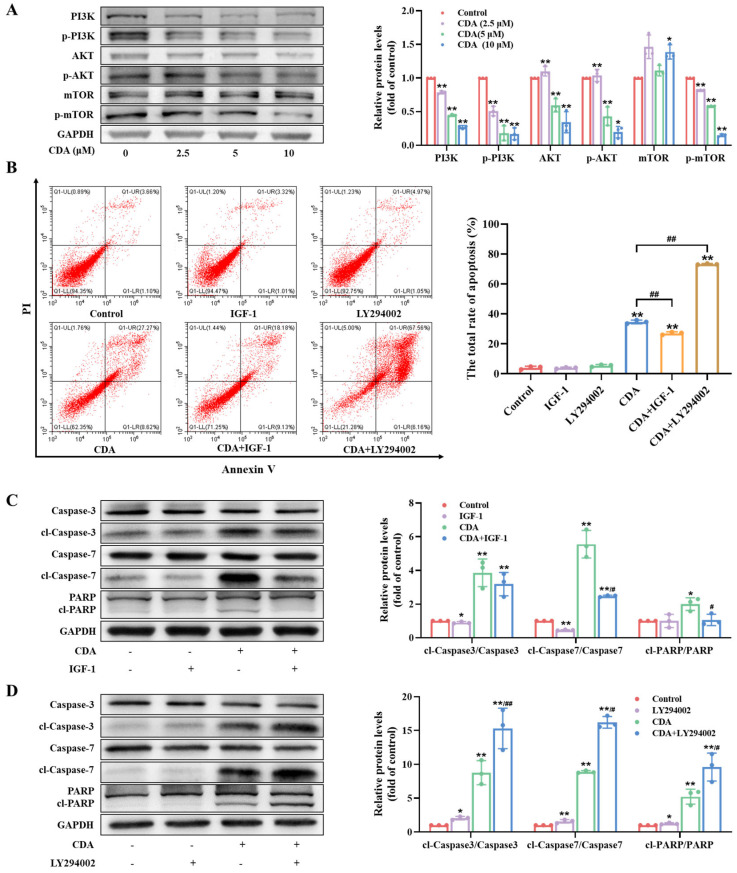
Curdepsidone A suppressed the PI3K/AKT pathway in HeLa cells. (**A**) HeLa cells were exposed to curdepsidone A for 48 h. The expression of PI3K/AKT pathway-related proteins was examined. (**B**–**D**) HeLa cells were pretreated with IGF-1 (200 ng/mL) or LY294002 (5 μM) for 2 h and then treated with curdepsidone A (10 μM) for 48 h. The apoptosis of HeLa cells (**B**) and the expression of caspase3, cl-caspase3, caspase7, cl-caspase7, PARP, and cl-PARP (**C**,**D**) were examined. Values are mean ± SD, *n* = 3, * *p* < 0.05, ** *p* < 0.01 versus the control group; ^#^ *p* < 0.05, ^##^ *p* < 0.01 versus the CDA group.

The PI3K/AKT signaling pathway plays a pivotal role in cancer development. To further uncover the mechanism behind the growth suppression of curdepsidone A on HeLa cells, we examined the effect of curdepsidone A on the PI3K/AKT pathway via Western blot. The results revealed that curdepsidone A could downregulate the levels of PI3K, p-PI3K, AKT, p-AKT, and p-mTOR in HeLa cells (Figure 5A), suggesting that curdepsidone A possibly induced apoptosis by hindering the PI3K/AKT pathway. Additionally, IGF-1 (200 ng/mL), the PI3K kinase activator (Figure S3), markedly withstood the percentage of apoptosis caused by curdepsidone A (Figure 5B). As shown in Figure 5C, pretreating HeLa cells with IGF-1 (200 ng/mL) for 2 h markedly decreased the expression level of cl-caspase3 and 7 and PARP. On the contrary, LY294002 (5 μM), the PI3K kinase inhibitor (Figure S3), could further promote the curdepsidone A-induced apoptosis (Figure 5B) and upregulated the curdepsidone A-induced cleavage of caspase3 and 7 and PARP (Figure 5D). To sum up, these results substantiated that the PI3K/AKT pathway participated in curdepsidone A-induced cell apoptosis.

### 2.8. Curdepsidone A Increased ROS Generation in HeLa Cells

**Figure 6:**
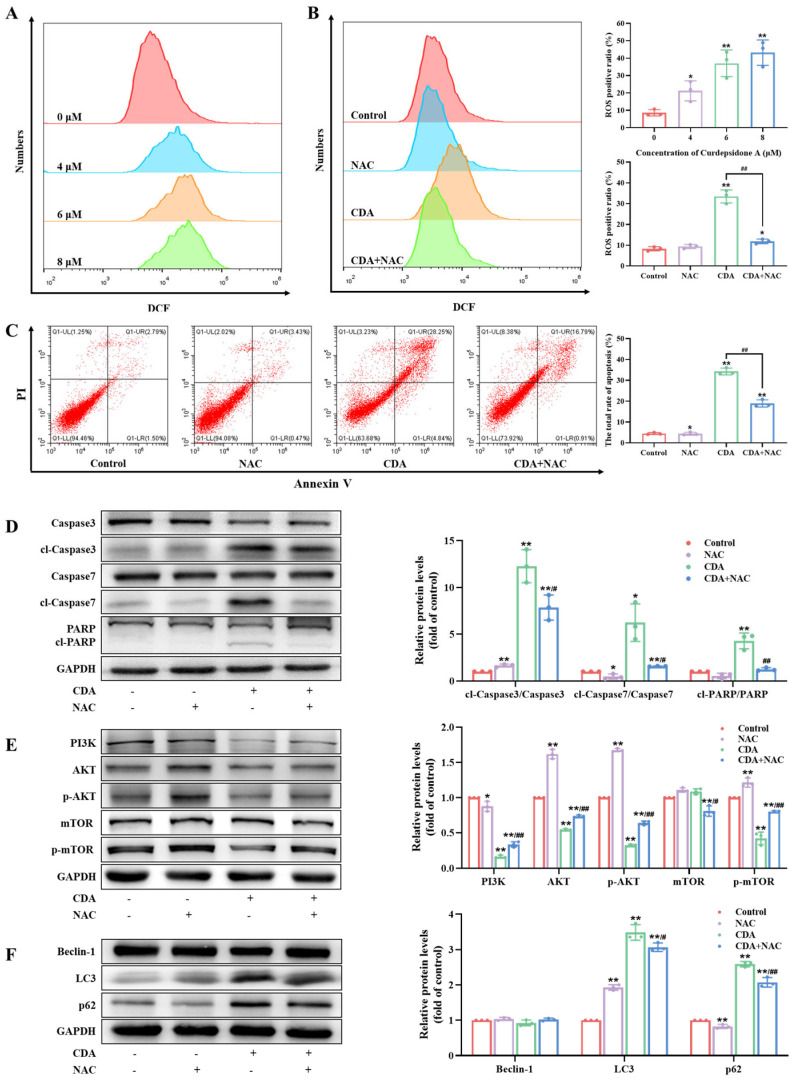
Curdepsidone A increased reactive oxygen species (ROS) generation in HeLa cells. (**A**) HeLa cells were exposed to curdepsidone A at concentrations of 0, 4, 6, and 8 μM for 24 h. Intracellular ROS levels were assessed. (**B**–**F**) HeLa cells were pretreated with NAC (5 mM) for 2 h and then treated with curdepsidone A (8 μM) for 24 h. Intracellular ROS levels (**B**), the apoptosis of HeLa cells (**C**), and the expression of caspase3, cl-caspase3, caspase7, cl-caspase7, PARP, cl-PARP (**D**), PI3K, AKT, p-AKT, mTOR, p-mTOR, Beclin-1, LC3 and p62 (**F**) were examined. Values are mean ± SD, *n* = 3, * *p* < 0.05, ** *p* < 0.01 versus the control group; ^#^ *p* < 0.05, ^##^ *p* < 0.01 versus the CDA group.

**Figure 7:**
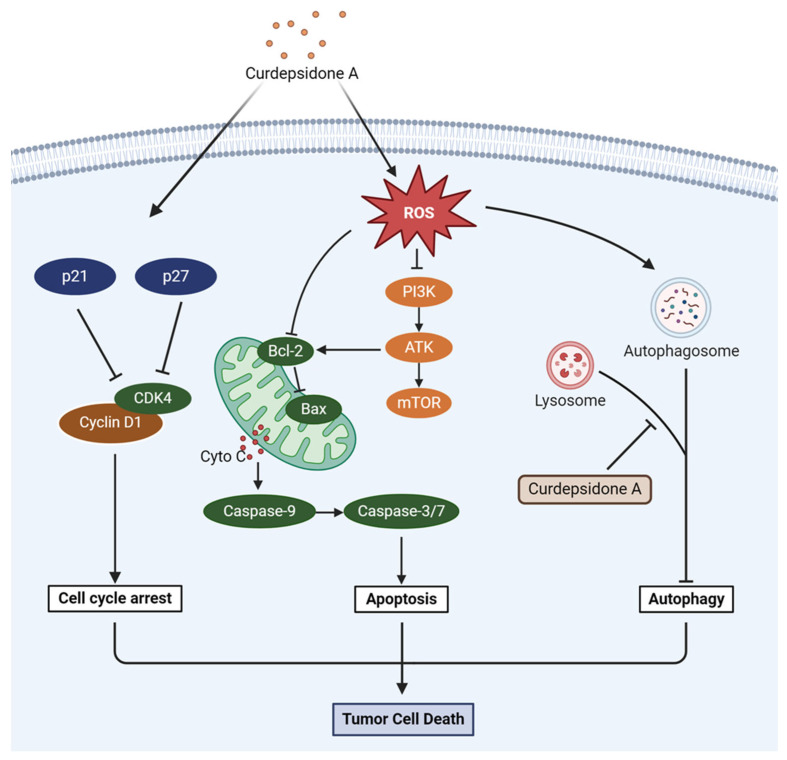
Curdepsidone A inhibited the growth of Hela cells.

The mitochondria metabolism can produce ROS and function as signaling molecules that are involved in numerous pathways [35]. Moreover, an excessive level of ROS can promote cancer cell death and inhibit cancer progression [36]. We evaluated the impact of curdepsidone A on ROS production in HeLa cells by using flow cytometry to measure the fluorescence of DCF. Curdepsidone A observably increased cellular ROS production in HeLa cells (Figure 6A). Then, we utilized NAC, a ROS scavenger, to clarify the role of ROS in curdepsidone A-induced cell apoptosis. The generation of ROS in HeLa cells generated by curdepsidone A was significantly reduced via pretreatment with NAC (5 mM) (Figure 6B). As shown in Figure 6C, compared to the CDA-only-treated group, the co-treatment of NAC and curdepsidone A considerably reduced apoptosis cell percentage. In addition, curdepsidone A attenuated the curdepsidone A-induced cleavage of caspase3 and 7 and PARP (Figure 6D). These results demonstrated that curdepsidone A induced cell apoptosis in HeLa cells via ROS generation. We then sought to explore whether the accumulation of ROS affected autophagy or the PI3K/AKT pathway. As shown in Figure 6E,F, it was proved that when HeLa cells were pretreated with NAC for 1 h, the expression of LC3 and p62 was obviously decreased, and the expression of PI3K, AKT, p-AKT, and p-mTOR was dramatically increased. Moreover, LY294002 and IGF-1 had no effect on ROS levels (Figure S5). Taken together, the results suggested that curdepsidone A could elevate intracellular ROS levels, intensifying the suppression of PI3K/AKT pathway and protective autophagy, ultimately leading to apoptosis in HeLa cells, as Figure 7 summarizes.

## 3. Discussion

Natural products play a crucial role in drug development [37]. Due to the unique environment of the ocean, which harbors abundant biodiversity, it serves as a significant source of natural compounds [38]. Hence, marine natural products are emerging as pivotal targets in the development of prospective new anticancer medications [39], and an increasing number of studies have reported that marine natural products can inhibit tumor development. Citromycin, isolated from the Antarctic marine-derived fungus *Sporothrix* sp., can inhibit the growth of ovarian cancer cells by suppressing the ERK1/2 pathway [40]. Holothurin A, isolated from *Holothuria scabra*, can inhibit EMT and the metastasis of prostate cancer through the suppression of the AKT/P38/JNK-MAPK pathway [41]. Depsidones exhibit various biological activities, including cytotoxicity, anti-inflammatory, and antimicrobial properties [11]. Himantormiones B, isolated from the Antarctic Lichen *Himantormia lugubris*, demonstrates potent cytotoxicity against colon cancer HCT116 cells [42]. Physodic acid, identified from the European lichen *Hypogymnia physodes*, can induce apoptosis in prostate cancer LNCaP cells [12]. In recent years, increasing evidence suggests that depsidones may possess potent anti-tumor activities. However, their mechanisms of action remain poorly understood. Curdepsidone A is a marine-derived derivative of depsidone and has shown promising inhibition of tumor cell proliferation in vitro [21]. This study aims to investigate the specific mechanisms of curdepsidone A underlying its anticancer effects in HeLa cells through in vitro experiments, providing a theoretical foundation for its potential candidacy as an anticancer drug. Our findings demonstrated that curdepsidone A could suppress cell growth, arrest cell cycle at the G0/G1 phase, obstruct autophagy flux, and promote endogenous apoptosis. In addition, curdepsidone A activated apoptosis by inhibiting the ROS-mediated PI3K/AKT pathway in HeLa cells.

Uncontrolled cell proliferation is one of the characteristics of cancer due to the disruption of the cell cycle [43]. As a result, regulating the cell cycle is becoming one of the most important means of cancer therapy. We observed that curdepsidone A could increase the proportion of G0/G1-phase cells, suggesting that curdepsidone A caused G0/G1-phase cycle arrest in HeLa cells. The activation of cyclin-dependent kinases (CDKs) is closely related to the dysregulation of the cell cycle mechanism [44]. CDK4/6-cyclin D1 complexes promote cell cycle progression and phosphorylate retinoblastoma protein (pRb) in response to growth signals [45]. P21 and p27, endogenous inhibitors of CDK inhibitors (CKIs), are in charge of preventing the cell cycle from progressing by suppressing CDKs [46,47]. In addition, both p21 and p27 are involved in several other pathways of cell metabolism, including DNA replication and repair, transcriptional regulation, and apoptosis [47,48]. We found that curdepsidone A suppressed the level of CDK4 and Cyclin D1 and promoted the level of p21 and p27. These findings showed that curdepsidone A suppressed the growth of HeLa cells by modulating the cell cycle-related proteins and triggering G0/G1-phase cell cycle arrest.

The most often dysregulated type of cell death in cancer is the endogenous mitochondrial pathway of apoptosis [49]. The main activation modes of endogenous apoptosis include mitochondrial damage, oxidative stress, endoplasmic reticulum stress, growth factor deprivation, DNA damage, and excessive mitosis [50]. When these stimuli converge on the mitochondria, the outer membrane of the mitochondria becomes more permeable, allowing cyto-c to be released into the cytoplasm, and MMP will be reduced. Subsequently, apoptotic bodies are formed, and apoptotic effector proteins like caspase3, 7, and 9 are activated [51]. PARP protein, a kind of DNA repair enzyme, is one of the most important substrates of caspase3. Cl-PARP is unable to repair DNA damage and eventually induces cell apoptosis. Curdepsidone A could reduce the MMP and raise the expression level of cytosolic cyto-c in HeLa cells. In addition, curdepsidone A activated caspase3, 7, and 9 and increased cleaved PARP levels. Thus, we inferred that curdepsidone A could induce apoptosis in HeLa cells via the mitochondria pathway.

Moreover, autophagy is a multi-step process that enables cells to resist various stresses and influences cell fate. It is possible to use abnormal autophagy as a tumor promoter to inhibit apoptosis or as a tumor suppressor to promote autophagy death [52]. It was reported that naringenin-induced autophagy promotes colon cancer cell survival [53]. However, dihydroartemisinin stimulated autophagy and induced cell death in cervical and endometrial cancer [54]. The process of autophagy involves a number of proteins. Beclin-1 is an autophagy regulatory protein that is associated with the formation and transport of autophagic vesicles. LC3 is an important protein located on the autophagosome membrane. It can bind to p62 proteins attached to ubiquitinated proteins, thereby wrapping the misfolded proteins into autophagosomes and forming autolysosomes for degradation [55]. While curdepsidone A did not affect Beclin-1 expression levels, it promoted the accumulation of LC3 and p62 proteins in HeLa cells, suggesting that curdepsidone A could block the autophagy flux in HeLa cells. GFP-RFP-LC3-adenovirus was used to further confirm the blocking effect of the downstream phases of autophagic flux induced by curdepsidone A, consistent with the effects of CQ. The combination of chemotherapeutic agents with autophagy inhibitors is also considered an effective anti-tumor strategy [56]. CQ was able to promote curdepsidone A-induced apoptosis by further activating Caspase7 and subsequent PARP cleavage, which might be due to enhanced autophagy flux blocking. These findings suggested that curdepsidone A promoted the apoptosis of HeLa cells through the inhibition of protective autophagy.

Abnormal activation of the PI3K/AKT pathway is common in human tumor cells. This process is stimulated in tumor cells to enhance their growth, survival, and metabolism, and the pathway is intimately related to autophagy [29,30]. Inhibiting the PI3K/AKT pathway, which is the upstream pathway of the anti-apoptosis protein Bcl-2, results in apoptosis via decreasing Bcl-2 expression [57]. Moreover, autophagy is inhibited by the PI3K α catalytic subunit, and autophagy gene expression is blocked by AKT via the activation of rapamycin complex 1 (mTORC1) [28]. In our study, curdepsidone A could inhibit the PI3K/AKT pathway, which was confirmed by adding the PI3K activator IGF-1 and inhibitor LY294002, respectively. These findings revealed that curdepsidone A-induced apoptosis in HeLa cells was regulated by the PI3K/AKT pathway.

ROS is one of the most important regulators in the cell signaling pathway [35]. Excessive ROS often inhibits tumor growth, causes irreversible damage to mitochondrial function, and induces the apoptosis of tumor cells [31,32]. Curdepsidone A raised the levels of intracellular ROS, caused the membrane of the mitochondria to become more permeable, leaked cyto-c into the cytosol, and induced the apoptosis of HeLa cells. In addition, we observed that the ROS scavenger NAC could reverse the blockade of autophagy flux and attenuate the apoptosis rate induced by curdepsidone A in HeLa cells. The PI3K/AKT pathway’s suppression caused by curdepsidone A was also prevented by NAC pretreatment. Meanwhile, neither the PI3K activator nor the inhibitor had any effect on the ROS level in HeLa cells. To sum up, our findings revealed that curdepsidone A induced apoptosis in HeLa cells partly through the activation of the ROS/PI3K/AKT axis.

In addition, we discovered that curdepsidone A dramatically decreased the total protein expression of PI3K and AKT without affecting the mRNA level (Figure S4). We conjectured that curdepsidone A might affect the protein synthesis pathway in HeLa cells. Further transcriptome and metabolomic analysis studies will be carried out to explore the underlying mechanism of curdepsidone A affecting protein level inhibition. Related research is being planned.

## 4. Materials and Methods

### 4.1. Reagents and Antibodies

Curdepsidone A was extracted from the secondary metabolites obtained from the rice solid fermentation of *Curvularia* sp. IFB-Z10. The purity of curdepsidone A (>95%) was determined via HPLC (Figure S1).

### 4.2. Cell Culture

The China Center for Type Culture Collection (CCTCC) provided the HeLa cell lines. HeLa cell lines were grown in 10% FBS (ExCell Bio, Suzhou, China) in DMEM medium (KeyGEN, Nanjing, China). The cells were kept in an incubator that was humidified with 5% CO_2_ at 37 °C.

### 4.3. Cell Viability Assay

After being seeded in 96-well plates for one night, cells (5 × 10^3^ cells/well) were exposed to various doses of tested samples. The original medium was removed after 48 h, and each well received fresh DMEM media containing 0.5 mg/mL of MTT. After incubating at 37 °C for 4 h, the original medium was replaced with DMSO to dissolve the formazan. The absorbance was determined with a microplate reader (Molecular Devices, Sunnyvale, CA, USA) at 570 and 630 nm.

### 4.4. Cell Proliferation Assay

Cell proliferation was measured by using the BeyoClick™ EdU Cell Proliferation Kit (Beyotime, Shanghai, China). In short, after exposing cells to various doses of curdepsidone A (0, 2.5, 5, 10 μM), cells were stained at 37 °C using 10 μM of EdU solution. Following that, the cells were fixed with 4% paraformaldehyde, rinsed with wash solution three times, treated with 0.3% Triton X-100, and then rinsed twice. Next, the solution for the Click reaction was added. After being incubated in a dark environment, the cells were three times rinsed with wash solution. Finally, the cell nucleus was dyed with Hoechst 33258 at 37 °C, after which the cells were investigated under a fluorescence microscope (Nikon, Tokyo, Japan).

### 4.5. Cell Cycle Analysis

For cell cycle detection, to ensure cell survival, we exposed HeLa cells to 0, 1, 2, and 4 μM of curdepsidone A. After a 24 h treatment with curdepsidone A, cells were harvested, cleaned, and fixed overnight at 4 °C. Whereafter, all samples were stained in the dark using propidium iodide (PI) and RNase (KeyGen, Nanjing, China). The cell cycle was examined by using a CytoFLEX flow cytometer (Backman Coulter, Brea, CA, USA).

### 4.6. Cell Apoptosis Analysis

To determine the percentage of apoptotic cells, the Annexin V-FITC/PI double-staining apoptosis detection kit (Elabscience, Wuhan, China) was utilized. In brief, after being treated with various doses of curdepsidone A, the cells were harvested and then stained in the dark with Annexin V-FITC and PI mixture in the binding buffer. A flow cytometer was used to identify the apoptotic cells.

### 4.7. MMP Evaluation

The cells were treated with curdepsidone A and then incubated at 37 °C with a JC-1 fluorescent probe (KeyGen, Nanjing, China). Following two rounds of washing with the incubation buffer, the cells were examined using a flow cytometer.

### 4.8. Examination of GFP-RFP-LC3B Punctuation

A GFP-RFP-LC3B adenoviruses kit (Hanbio, Shanghai, China) was used to examine GFP-RFP-LC3B punctuation. As directed by the manufacturer, HeLa cells were cultured in 12-well plates with coverslips before being transiently transfected with GFP-RFP-LC3B adenoviruses. After a 6 h period, cells were incubated with curdepsidone A for 24 h and fixed with 4% formaldehyde. The results were examined using a confocal microscope (Nikon, Tokyo, Japan).

### 4.9. Measurement of Intracellular ROS Generation

A DCFH-DA probe (Beyotime, Shanghai, China) was used to determine the levels of intracellular ROS. After treatment with curdepsidone A for 24 h, cells were laden with 10 μM DCFH-DA at 37 °C. Subsequently, the cells were gathered, suspended in PBS, and promptly quantified with a flow cytometer.

### 4.10. Western Blot Analysis

After being exposed to curdepsidone A, HeLa cells were collected and lysed in RIPA buffer (Biotech Well, Shanghai, China) on ice. After quantified with the BCA protein assay (Elabscience, Wuhan, China), each sample was added to 5 × loading buffer (Beyotime, Shanghai, China) and then heated at 100 °C to denature the protein. The proteins were electrotransferred to PVDF membranes (Millipore, Billerica, MA, USA) after the lysate proteins were separated via SDS-PAGE. The membranes were hybridized with the suitable primary antibodies in the blocking solution for an entire night at 4 °C after blocking for 2 h. After that, the membranes underwent three TBST washes, one hour of incubation at 37 °C with a suitable secondary antibody, and another three TBST washes. Lastly, the ECL Chemiluminescence Kit (Beyotime, Shanghai, China) was used to detect protein signals on BG-gdsAUTO 720 (Baygene, Beijing, China). The expression of GADPH is regarded as a loading control. The primary antibodies against p-PI3K and p-mTOR were purchased from Cell Signaling Technology. Caspase3, cl-caspase3, caspase7, cyto-c, Apaf-1, LC3, PI3K, and mTOR were purchased from Abcam. GAPDH, CDK4, Cyclin D1, PARP, Bcl-2, Bax, Caspase9, Beclin-1, p62, AKT, and p-AKT were purchased from Proteintech.

### 4.11. Statistical Analysis

The mean standard deviation (±SD) was used to express every data set. Analysis of variance (ANOVA) was performed using GraphPad Prism 8.0 (GraphPad Software, San Diego, CA, USA). *p*-values were deemed statistically significant if they were less than 0.05.

## 5. Conclusions

In summary, we verified that curdepsidone A, a kind of bioactive compound of marine microorganic origin with developing potential, could suppress the proliferation of cervical cancer HeLa cells by triggering G0/G1-phase cell cycle arrest and apoptosis. Curdepsidone A promoted apoptosis by activating ROS production, suppressing the PI3K/AKT pathway, and inhibiting protective autophagy. Based on our findings, curdepsidone A was expected to become a candidate drug for the treatment of cervical cancer.

## Figures and Tables

**Table 1:** IC_50_ values of curdepsidone A towards different cancer cell lines.

Cell Line	IC_50_ of Curdepsidone A (μM)
HeLa	6.28 ± 0.38
BEL-7402	17.37 ± 1.84
HepG2	8.17 ± 0.42
K562	16.76 ± 0.18
SW1116	8.85 ± 1.50
MCF-7	15.37± 2.10
MGC803	12.09 ± 1.59

## Data Availability

The data supporting the findings of this study are available from the corresponding author upon reasonable request.

## Supplementary Materials

The following supporting information can be downloaded via this link: https://www.mdpi.com/article/10.3390/md22050227/s1, Figure S1: The purity of curdepsidone A; Figure S2: The viability of different tumor cells; Figure S3: The effects of IGF-1 and LY294002 on the curdepsidone A-induced inhibition of the PI3K/AKT pathway; Figure S4: The mRNA levels of PI3K and AKT; Figure S5: The effect of IGF-1 and LY294002 on the curdepsidone A-induced upregulation of ROS levels; Table S1: Primers used in this study.
