# Supplementary material for: Curdepsidone A Induces Intrinsic Apoptosis and Inhibits Protective Autophagy via the ROS/PI3K/AKT Signaling Pathway in HeLa Cells
Source: Mar Drugs. 2024 May 17;22(5):227. doi: 10.3390/md22050227 (PMC11123476; doi:10.3390/md22050227)
Supplement: Supplementary file 1 [file marinedrugs-22-00227-s001.zip › marinedrugs-2982257-supplementary.pdf]

Supplementary Materials

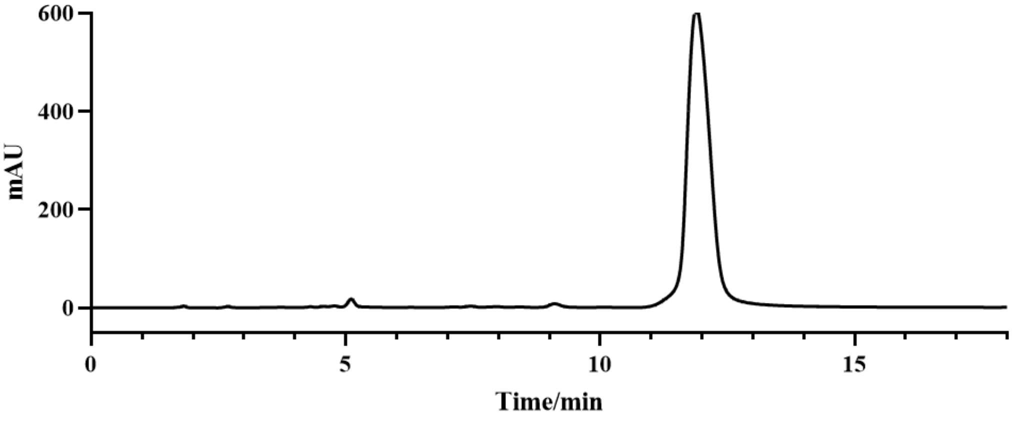

Figure S1. The purity of curdepsidone A.

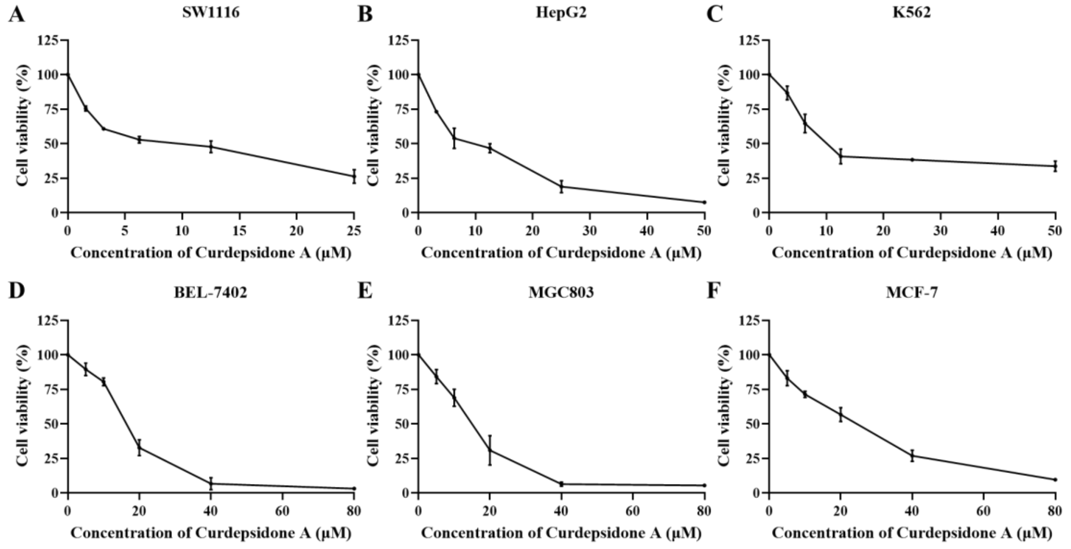

Figure S2. The viability of different tumor cells. (A-F) SW1116 (A), HepG2 (B), K562 (C), BEL-7402 (D), MGC803 (E), and MCF-7 (F) cells were exposed to various concentrations of curdepsidone A for 48 h. Cell viability was analyzed by MTT assay. Values are mean ± SD, n = 3, *$P < 0.05$. **$P < 0.01$.

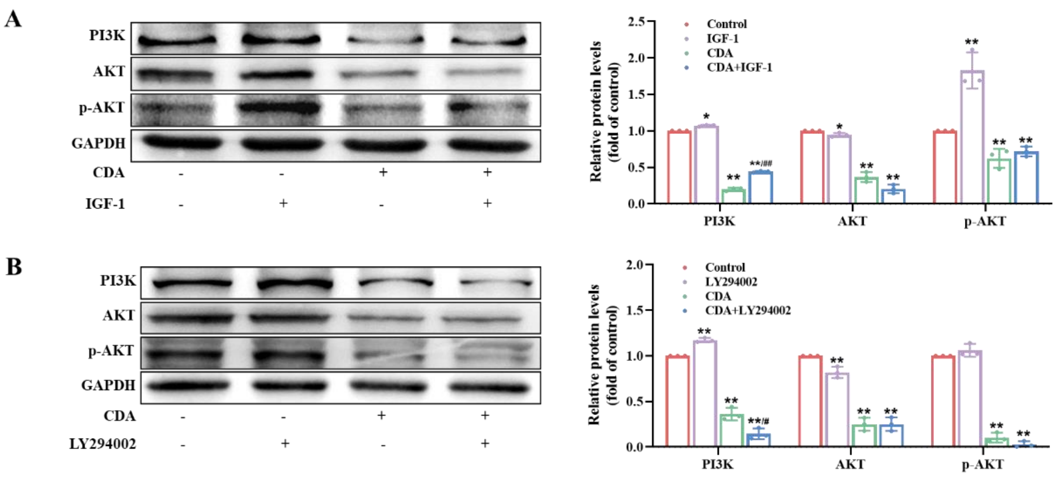

Figure S3. The effect of IGF-1 and LY294002 on curdepsidone A-induced inhibition of PI3K/AKT pathway. (A-B) Hela cells were pretreated with IGF-1 (200 ng/mL) (A) or LY294002 (5 μM) (B) and then treated with curdepsidone A (10 μM) for 48 h. The expression levels of PI3K, AKT, and p-AKT were examined. Values are mean ± SD, n = 3, **P* < 0.05, ***P* < 0.01 versus control group; #*P* < 0.05, ##*P* < 0.01 versus CDA group.

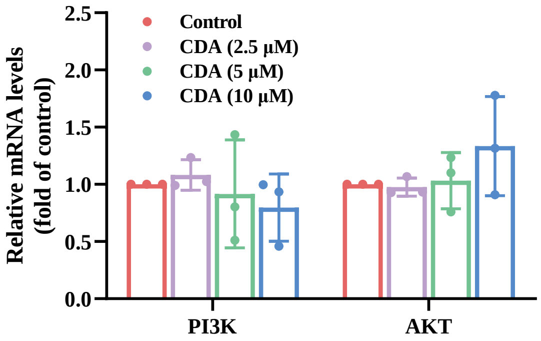

Figure S4. The mRNA levels of PI3K and AKT. Hela cells were exposed to 2.5, 5, and 10 μM curdepsidone A for 48 h. The mRNA levels of PI3K and AKT were examined by qPCR.

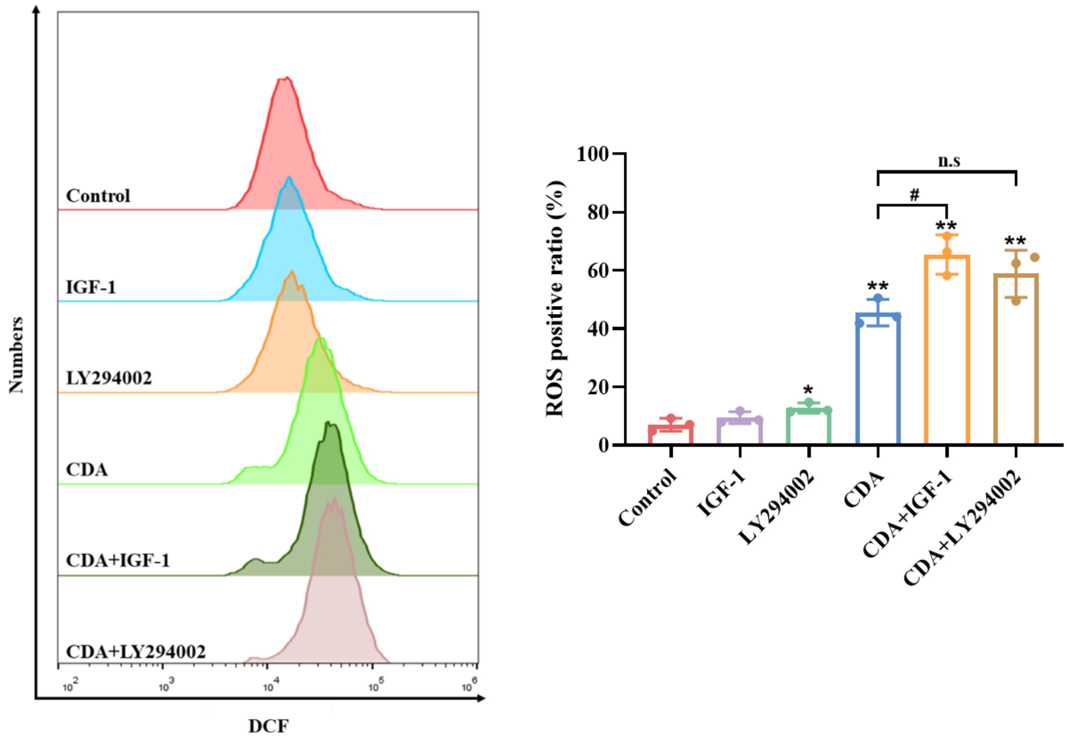

Figure S5. The effect of IGF-1 and LY294002 on curdepsidone A-induced upregulation of ROS levels. Hela cells were pretreated with IGF-1 (200 ng/mL) (A) or LY294002 (5 μM) (B) and then treated with curdepsidone A (10 μM) for 24 h. Intracellular ROS levels were assessed by flow cytometry.

Table S1. Primers used in this study.

**Primer**	**Sequence (5'-3')**
AKT-F	AAGCACCGCGTGACCATGAA
AKT-R	GGCCTGTGGCCTTCTCCTTC
PI3K-F	CGGTTCCGCCAGTGTTGTGA
PI3K-R	GCCCTGCAGTCAACATCAGC
GAPDH-F	GCGGGGCTCTCCAGAACATC
GAPDH-R	TCCACCACTGACACGTTGGC
